# Determinants of Health Care Services Utilization among First Generation Afghan Migrants in Istanbul

**DOI:** 10.3390/ijerph14020201

**Published:** 2017-02-17

**Authors:** Qais Alemi, Carl Stempel, Patrick Marius Koga, Valerie Smith, Didem Danis, Kelly Baek, Susanne Montgomery

**Affiliations:** 1 Department of Social Work & Social Ecology, School of Behavioral Health, Loma Linda University, 1898 Business Center Drive, San Bernardino, CA 92408, USA; kbaek@llu.edu (K.B.); smontgomery@llu.edu (S.M.); 2 Department of Sociology and Social Services, California State University, East Bay, 25800 Carlos Bee Blvd., Hayward, CA 94542, USA; carl.stempel@csueastbay.edu; 3 Department of Public Health Sciences, UCD School of Medicine, University of California, One Shields Avenue, Med Sci 1-C, Davis, CA 95616, USA; pmkoga@ucdavis.edu; 4 Department of Health Sciences, California State University, East Bay, 25800 Carlos Bee Blvd., Hayward, CA 94542, USA; valeriejs2@gmail.com; 5 Department of Sociology, Galatasaray University, Istanbul 34349, Turkey; didemdanis@yahoo.com

**Keywords:** Afghan, Andersen Model, health services, medications, migrant, Turkey, utilization

## Abstract

There is insufficient empirical evidence on the correlates of health care utilization of irregular migrants currently living in Turkey. The aim of this study was to identify individual level determinants associated with health service and medication use. One hundred and fifty-five Afghans completed surveys assessing service utilization including encounters with primary care physicians and outpatient specialists in addition to the use of prescription and nonprescription medicines. Multivariate logistic regression analyses were employed to examine associations between service use and a range of predisposing, enabling, and perceived need factors. Health services utilization was lowest for outpatient specialists (20%) and highest for nonprescription medications (37%). Female gender and higher income predicted encounters with primary care physicians. Income, and other enabling factors such as family presence in Turkey predicted encounters with outpatient specialists. Perceived illness-related need factors had little to no influence on use of services; however, asylum difficulties increased the likelihood for encounters with primary care physicians, outpatient services, and the use of prescription medications. This study suggests that health services use among Afghan migrants in Turkey is low considering the extent of their perceived illness-related needs, which may be further exacerbated by the precarious conditions in which they live.

## 1. Introduction

Of the 1.26 million new asylum claims made in European Union (EU) countries at the end of 2015, 176,000 were made by Afghans, ranking just second to claims made by Syrians [1]. The migration of Afghans to the EU has increased dramatically in recent years due to socio-economic challenges and a prolonged conflict in Afghanistan, in addition to deteriorating economic conditions and policy shifts toward existing (undocumented) Afghan refugees in Iran and Pakistan [2] that result in resettling to other regions by these migrants. Turkey continues to be a major transit hub for Afghans seeking to reach EU countries [3], but also a place of permanent residence for some, as evidenced by thousands of asylum claims lodged by Afghans in recent years [4]. 

However, Turkish asylum and settlement laws grant protection only to refugees from Europe and to individuals of Turkish descent, respectively [5], which marks the presence of Afghans in Turkey as irregular. It appears that Turkey has recently recognized the importance of creating a coherent migration management framework. Adopted in 2013 and implemented in 2014 by the Turkish Parliament, the Law 6458 on Foreigners and International Protection (*Yabancılar ve Uluslararası Koruma Kanunu*) is intended as a step toward managing both legal and non-formal migration to Turkey [6]. Under this Law’s Article 96, the mutual “adaptation” of immigrants and society is supposed to be facilitated through courses and information campaigns, depending on available funds and stakeholders’ recommendations.

Nevertheless, until this law applauded by the United Nations (UN) and EU is fully shared with all stakeholders and enforced, the predicament of the status quo remains, with all its adverse effects on migrant health.

Post-migration stressors (e.g., asylum difficulties or administrative difficulties in the application process, employment-related problems, etc.) associated with their insecure status as irregular migrants in Turkey have led to high rates of psychological distress, as demonstrated recently by the authors of this study [7]. The situation of Afghans in Turkey, however, remains understudied, and the significance of further investigating their health needs rests on several factors, including: (1) their continual migration to Turkey; (2) health needs that may transcend psychological help for traumatic symptoms to include non-communicable diseases such as hypertension and chronic pain commonly observed in refugees [8]; and, (3) the fact that unmet health needs are likely exacerbated by difficulties in accessing health services due to their legal status. 

Despite these needs, we found no studies of access to and use of health services among irregular/undocumented migrants or asylum-seekers in Turkey. Previous work has focused on migrants in the EU where access to health services for irregular migrants may be relatively poor partly due to linguistic and cultural barriers [9], the fact that few countries grant access to care beyond emergency services, and because migrants often experience fears of being reported to police and immigration authorities by health care staff [10]. A later review confirms these access barriers among asylum-seekers, and additionally highlights the inability to pay for services, discrimination on the part of health care staff, and difficulties in navigating health services (improved through social/community supports) as access barriers [11]. 

In contrast, Hadgkiss and Renzaho cite various studies showing that asylum seekers’ utilization of general practitioners/primary care services and nurses is high (ranging from 55% to 73% report using such services), with prescription of medication being a common outcome of such encounters. Specifically, Gerritsen et al. [12] showed that Afghans in the Netherlands report higher contacts with a general practitioner (GP) and more medication (analgesics, sleeping pills) use than a Somali comparison group, and, that female gender, older age, and perceived (poor) general health status predict health service use. As opposed to the Netherlands where health services are more easily accessible for irregular migrants, regardless of their nationality [10], in Turkey irregular or undocumented migrants are not entitled to any public health services. Instead, only Syrians, who are under “temporary protection” status, are legally covered by Turkey’s public health care system [13]. Asylum-seekers from other countries of origin (international protection applicants) do not automatically qualify for coverage, and as a general rule can only utilize health services in the province where they are registered and required to reside. Registration is processed with provincial DGMM Directorates (Directorate General of Migration Management), which under normal circumstances ensures international protection applicants free-of-charge access to primary (e.g., health centers, tuberculosis dispensaries), secondary (state hospitals), and tertiary (research and training hospitals) health services. Provincial DGMMs provide international protection applicants a Foreigners Identification Number (FIN), which healthcare providers use for intake and processing purposes; therefore, with the exception of emergency health services that can be spontaneously accessed at any time, without a FIN irregular migrants cannot access the full range of services [14]. 

This paper aims to explore the extent to which migrant Afghans residing in Istanbul, Turkey use a variety of health services. These services include encounters with primary care physicians (PCPs), outpatient (OP) specialists, and the use of prescription as well as nonprescription medications—used as proxies for health services use, as shown in a recent large-scale epidemiological study of EU migrants [15]. This paper also aims to identify the factors associated with the use of health services, as guided by Andersen’s Behavioral Model of Health Services Use or the “Andersen Model” [16]. 

Health services utilization, according to the Andersen model, is a function of three components: (1) predisposing factors, which may include demographic characteristics (e.g., age, gender); (2) enabling factors or conditions that make health service resources available to an individual (e.g., income, access to a regular source of care); and (3) perceived (e.g., quality of life, symptoms) as well as evaluated (e.g., diagnoses) need factors, which are described as the most immediate reason for health services use to take place. This model has been widely applied to studies of migrant populations, notably by Laban et al. [17], who used Andersen’s model with a sample of Iraqi asylum-seekers in the Netherlands where it was hypothesized that a special set of need variables, including those reflecting post-migration living difficulties (e.g., long asylum application procedures) would predict service use.

Results showed that long asylum procedures did not predict preventive service use, but did predict mental health service use and prescription drug use only, possibly due to the stress associated with long asylum processes. Moreover, this study cites that other need variables, including low perceived quality of general health and functional disabilities, were the most important predictors of use. We tested similar relationships here, and hypothesized that health service use would be predicted by various predisposing and enabling factors, and that the effects of (perceived) need factors, including health and mental health measures and a range of post-migration living difficulties, would the most important predictors of use.

## 2. Materials and Methods

### 2.1. Participants and Procedures

The inclusion criteria for this cross-sectional study was limited to migrant adults over the age of 18 who are of Afghan ancestry and currently reside in Istanbul. The ethical merits of this study were reviewed and approved by the first author’s affiliated university Institutional Review Board (IRB), with approvals also obtained locally through Galatasaray University in Istanbul (Ethical approval code: 5150291). An IRB-approved information sheet explaining participants’ rights and the survey were used for soliciting participants using a combination of convenience, snowball, and street-intercept sampling techniques in Istanbul’s Zeytinburnu district over a 10-day recruitment period in September 2015. The first author of this study along with a research assistant from a local university in Istanbul, both fluent in Dari, explained the survey to potential participants as an effort to document their health and life experiences. Then participants completed the survey on their own on sidewalks, in local cafés, public venues such as parks, in local shops where they worked, and in their residences (a few participants with literacy challenges were interviewed).

### 2.2. Measures

Surveys were completed in Dari, which is a language commonly spoken among Afghans. With the exception of the Afghan Symptom Checklist (ASCL), in which a Dari version was already available (courtesy of Ken Miller), all other survey items and scales, including the SF-8 and Post-Migration Living Difficulties (PMLDs) checklist went through a rigorous translation-back translation process with assistance from members of the Afghan refugee community in Southern California.

#### 2.2.1. Predisposing Factors

Surveys assessed information on age, gender, marital status (1 = currently married, 0 = unmarried (includes never married, divorced/separated, widowed)), and educational attainment (1 = post-secondary and beyond, 0 = secondary school and lower).

#### 2.2.2. Enabling Factors

Surveys included questions on length of residence in Turkey (1 = ≥1 year, 0 = <1 year) while a question on family presence in Turkey provided indication of social capital. Socio-economic (SES) variables included questions on employment status (1 = employed, 0 = unemployed, including retired and disabled) and income stability (using a binary “yes/no” response assessing whether respondents are able to meet monthly financial needs with current income, 1 = stable, 0 = unstable). We also asked participants whether they had access to a regular source of health care using a binary “yes/no” response choice (1 = access, 0 = no access); however, this question did not refer to a specific source of health care.

#### 2.2.3. Need Factors (Perceived)

##### Health-Related Quality of Life (HR-QoL)

The physical and mental components of the Short-Form/SF-8 [18] were used for measuring HR-QoL. Items included in the physical health component related to: physical functioning (presence and extent of physical limitations), role limitations (due to physical health problems), bodily pain, and overall general health. The mental health component contained four items related to vitality (subjective account of energy level), social functioning, role limitations owing to emotional problems, and mental health (extent of emotional problems). Items were based on a four-week recall period with each item consisting of a five or six-point response choice. A norm based scoring procedure was used in order to generate Physical (PCS) and Mental (MCS) Component Summary scores by weighting each health domain scale score and computing aggregate scores for each measure as indicated in the instrument’s guidelines [19]. Both subscales demonstrated acceptable scale reliability (PCS: *Cronbach’s α* = 0.75; MCS: *Cronbach’s α* = 0.70). Additionally, we established convergent validity as individual items in the PCS and MCS were strongly correlated with their respective component summary measures (PCS item range: Pearson’s r = 0.63–0.82; MCS item range: Pearson’s r = 0.51–0.090).

##### Psychological Distress Symptoms

The frequency of distress symptoms was measured using a culturally-grounded measure of mental health, the Afghan Symptom Checklist or ASCL [20]. The ASCL consisted of 23 items familiar to both western psychiatry and Afghan society (e.g., “asabi”/high stressed, difficulty concentrating, sadness). Each question asked the participant to think back on the previous two weeks. Scores ranged from 23 to 115 derived from response choices ranging from “1” (“never”) to “5” (“everyday”). The ASCL demonstrated excellent reliability in this sample (*Cronbach’s α* = 0.942).

##### Post-Migration Living Difficulties (PMLDs)

The PMLD checklist [21] was used for measuring the severity of post-migration problems commonly encountered by asylum-seekers within the past 12 months. Responses were rated on a five-point Likert-type scale ranging from “0” (“no problem at all”) to “4” (“a very serious problem”). In a previous paper based on this sample [6], the authors identified five subscales through principal components analysis (PCA) (their respective Cronbach’s alphas ranging from 0.719 to 0.891). These five subscales were labeled (1) “conditions of extreme precarity” (nine-items: fears of being sent home, loneliness, poverty, etc.); (2) “asylum difficulties” (four-items: delays in processing asylum applications); (3) “employment-related problems” (three-items: e.g., no permission to work); (4) “access to medical and social services” (four-items: e.g., little help from charities), and (5) “marginalization and family-related stressors” (four-items: e.g., worries about family back home, communication difficulties/language barriers).

#### 2.2.4. Health Service Use (Dependent Variable)

Informed by previous studies examining service use among Afghans and other ethnicities residing in the EU [12,17], we asked participants whether they accessed any of the following health services within specified time-frames with a dichotomous “yes/no” response choice. Services included encounters with PCPs (past two months), and outpatient (OP) specialists (past two months), as well as the use of prescription (past 14 days) and nonprescription medicines (past 14 days). 

### 2.3. Data Analysis

SPSS, version 23.0 (SPSS Inc., Chicago, IL, USA) was used for all data analysis. First, we replaced missing scores for scales with less than 10% missing items by using the average of completed scale items. Bivariate tests were used to examine associations between the covariates (predisposing, enabling, and need factors) and the health service use outcomes (PCPs, OP specialists, prescribed medicines, un-prescribed medicines), where chi-square tests of independence and independent samples *t*-tests were applied as appropriate. We subsequently entered variables into multivariate logistic regressions to identify factors predictive of health service use. For a consistent set of parsimonious multivariate models, variables were selected based on their conceptual relevance, as informed by previous research, and their statistical significance (*p* < 0.05) at the bivariate level. For each predictive model, in the first step we entered predisposing factors (gender); next, we entered enabling factors (length of residence in Turkey, family presence in Turkey, income, access to a regular provider), followed by perceived need factors (PCS and MCS indices, ASCL scores, and scores for PMLD subscales). Multivariate logistic regression analysis computed odds ratios (ORs) with 95% confidence intervals and *p*-values of the association between each covariate and use of health services. Additionally, multiple regressions were carried out to screen for multicollinearity through an examination of tolerance statistics for predictor variables in each model. Tolerance was found to be greater than 0.1 for all variables, indicating that multicollinearity was not a problem. Linearity of the logit was ascertained by ensuring that *p*-values for Hosmer and Lemeshow tests for each model exceeded 0.05, which we confirmed. Statistical significance was considered at *p* < 0.05; however, given the sample size, relationships approaching significance at *p* < 0.10 are reported as well.

## 3. Results

### 3.1. Socio-Demographic Characteristics

Table 1 shows that a sample of 155 Afghans participated in this study who were on average 25.4 ± 7.7 years of age, and had resided in Turkey for over two years (2.49 ± 3.35, range: <1 year to 18 years). The majority of participants were male (84.3%), unemployed (78.4%), with unstable income (75%), and only one-third had access to a regular source of health care. Physical health status/PCS scores (43.38 ± 9.91), and mental health status/MCS scores (38.87 ± 10.57) were well below indices observed in the Turkish general population [22], indicating poor HR-QoL. ASCL scores (59.81 ± 21.52) suggest high rates of psychological distress with symptoms of sadness, *“jigar khuni”* (reaction to a painful experience), “thinking too much”, concentration difficulty, and hopelessness occurring most often in the past two weeks. PMLDs occurred at moderately high rates with PMLD-3 (employment-related problems) rated as most severe, followed by PMLD-5 (marginalization and family-related stressors). 

With respect to health services utilization, the use of nonprescription medicines was highest (*n* = 58, 37.4%), followed by use of prescription medications (*n* = 40, 25.8%). Preventive services such as encounters with a PCP (*n* = 34, 21.9%) occurred slightly more than encounters with outpatient specialists (*n* = 31, 20%) in the past two months.

### 3.2. Bivariate Associations with Health Services Use

Bivariate tests (not tabled) reveal that no significant relationship was observed between the predisposing factors and any of the health services use variables; however, the relationship between female gender and PCP use approached significance (*p* < 0.10). Among the enabling factors, higher income and having access to a regular source of health care were associated with all services, except nonprescription medication use. Additionally, nonprescription medication use increased significantly with employment, as did OP specialist use. Of the need factors, associations varied across the service types; for example, lower physical and mental health status was associated with nonprescription medication use. Lower severity of PMLDs-1 (conditions of extreme precarity), -3 (employment-related problems), and -5 (marginalization and family-related stressors) significantly increased encounters with PCPs and OP specialists. 

### 3.3. Predictors of Health Services Use

#### 3.3.1. Encounters with PCPs (Past Two Months)

Multivariate analyses are described in Table 2. Encounters with PCPs are significantly associated with female gender in step 1 of the model (consisting of predisposing factors). Gender remains significant in step 2 after entering enabling factors, of which higher income is a significant predictor of use. Upon entering perceived need factors in step 3, the effect of gender and income persist while need factors including higher precarity (PMLD-1) decreased the likelihood of encounters with PCPs (this relationship approaches significance), whereas higher severity of asylum difficulties (PMLD-2) significantly increased the likelihood of encounters with PCPs. 

#### 3.3.2. Encounters with OP Specialists (Past Two Months)

In terms of encounters with OP specialists (see Table 2), no significant relationship was observed between gender and OP use in step 1; however, in step 2, enabling factors such as higher income significantly increased the likelihood of use. Also, having family present in Turkey and access to a regular source of care, while increasing the likelihood of use, were marginally significant. However, in step 3 the effect of income was no longer significant, whereas the effect of family presence emerged as significant; in fact, the OR for family presence increased from 2.685 (in step 2) to 4.883 (in step 3). Among the need factors, higher severity of distress symptoms significantly decreased the likelihood of use. However, higher severity of asylum difficulties (PMLD-2) significantly increased the likelihood of encounters with OP specialists, while higher employment-related problems (PMLD-3) decreased use (this relationship approached significance).

#### 3.3.3. Use of Prescription Medications (Past 14 Days)

As shown in Table 3, when we explored prescription medication use, in step 1 no significant association was observed between gender and use. This (non-significant) relationship carried over after entering enabling factors in step 2. Of the enabling factors, family presence in Turkey, higher income, and having regular access to a provider significantly predicted use of prescription medications. When need factors were entered in step 3, these associations somewhat persisted as family presence remained significant and income approached significance. Of the need factors, higher severity of asylum difficulties predicted use. Additionally, lower distress symptoms predicted use; however, this relationship approached significance.

#### 3.3.4. Use of Nonprescription Medications (Past 14 Days)

When we explored non-prescription medication use, in step 1 no significant association was observed between gender and use. This (non-significant) relationship carried over consistently across all models. No enabling factors predicted non-prescription medication use. When need factors were entered in step 3, only distress symptoms significantly predicted use; however, this relationship was marginally significant. 

## 4. Discussion

Among a community-based sample in Istanbul, this study aimed to explore the extent to which first generation Afghan migrants use a variety of health services, and secondly to investigate factors associated with service use, as guided by the Andersen Model. Overall, our data suggests that the frequency of health service use among Afghan migrants residing in Turkey is quite low when compared to rates observed among asylum-seekers in the EU [11], which provides the closest comparison possible, given the sparse literature on (irregular and asylum-seeking) migrants’ access to health services in Turkey. In this study, we found that encounters with OP specialists (20%) were lowest, PCP use slightly higher, and medication use, namely nonprescription medications (37%), was highest. The low usage rates could be a result of our sample being predominantly composed of young men, who have been shown to use preventive health services less frequently than women or older men [12]. Moreover, women’s higher use of preventive services observed here may be due to the greater need for PCPs that provide support and consultations for various types of health screenings and prenatal care.

As expected, family presence in Turkey, income, and having access to a regular source of care positively correlate with use of all services other than medication (prescription and nonprescription) use at the bivariate level, and to some degree in multivariate analyses after adjusting for need factors. While income may certainly provide individuals with the means of covering out-of-pocket health care costs in Turkey, having access to a regular source (a gatekeeper), and family facilitates linkages to more specialized OP services, which concurs with previous findings [23,24]. Also, other enabling factors could be operating here, unaccounted for in our survey, such as where Afghans reside in Turkey (Zeytinburnu—a large resettlement hub in Istanbul) and the assistance from fellow Afghans familiar with navigating local health clinics. Future research is needed to identify and characterize these and other access-enabling channels that Afghans depend on.

In sum, having personal and social resources are so critical to the use of health services, that these factors may override the influence of physical and mental health problems observed in our sample. It is important to note that despite the low HR-QoL indices and high psychological distress levels observed here, these perceived illness factors had little to no influence on service use as hypothesized, contrasting with previous research [17]. The reason for this unexpected result is not entirely clear; however, it may indicate an unmet health need further exacerbated by their precarious situations and structural barriers that restrict Afghans in need of care from accessing professional health services. This hypothesis is supported by the greater reliance on nonprescription medications (e.g., over-the-counter analgesics) for physical health problems.

Moreover, our data suggests that linguistic and discrimination-related factors deter the use of health services, as demonstrated in the inverse bivariate relationship between PMLD-5 stressors and use of PCPs and OP specialists. This aligns with a recent review of asylum-seekers in the EU [11] indicating that factors such as discrimination on the part of health care provider and difficulty navigating health care systems serve as barriers to health care use. However, the effect of PMLD-5 did not persist in multivariate analyses, and strikingly PMLD-2 emerged as a significant predictor of service use (except for nonprescription medications). The positive association between PMLD-2 and service use is noteworthy because this implies that individuals facing asylum difficulties are meeting with government or charity agencies who are connecting them to services. Thus, asylum difficulties, though stressful in this population [7], may be reflecting a kind of social capital and would be better classified as an enabling factor for health care utilization, or a mixture of need and enabling, which future research may examine. 

A strength of our study was that it is among the first papers to examine health care utilization patterns among irregular migrants in Turkey. Nevertheless, the study has several important limitations. The small and non-random sample limits generalizability, and female participants were underrepresented; however, this may reflect actual migration trends among Afghans which favors males. Nonetheless, future research with Afghans and other irregular migrant populations in Turkey should place strong emphasis on pregnant and childrearing women, a critical period for both child and mother. Second, we assessed health services use based on self-report data rather than using medical records. Also, we did not account for use of emergency department (ED) services in our analysis, a major portal by which irregular migrants seek health care services. Future research ought to consider this either as an outcome variable or as an enabling factor given that ED encounters may result in referrals to other services. Lastly, the cross-sectional design does not allow for cause-effect relationships to be established. We suggest future longitudinal research to further explore health care services use among this population, which may change for some who build stronger social networks, learn to better navigate the health system, and gain some degree of socio-economic stability needed to pay for out-of-pocket costs for services.

## 5. Conclusions

In conclusion, this study shows health service use is low among Afghan migrants in Istanbul, and that service utilization is determined by a number of factors, namely having personal and social resources. The most striking finding was that perceived illness level had limited influence on service use. Taken together, these observations have implications for health policies that expand entitlements to migrants in precarious situations, instead of recognizing access to health care as a mere privilege [25]. An additional implication is that health policies should mitigate factors that pose barriers to seeking health care by improving language and cultural services (e.g., providing interpreters) and migrants’ health literacy, as well as by improving health worker competencies and addressing their negative perceptions toward migrants [26]. We hope that in the implementation of the above-mentioned Law 6458 on Foreigners and International Protection, the findings from this study will inform Turkish health policies towards Afghans who, as shown here and in our previous research, are at great risk of health and psychosocial problems. 

## Figures and Tables

**Table 1:** Socio-demographic characteristics (*n* = 155).

Factors	*n*	%	M (SD)
Age, years **^a^**			25.4 (7.7)
Gender			
Female	24	15.7	
Male	129	84.3	
Education			
≤HS (High School) Diploma	126	83.4	
≥College	25	16.6	
Length of Residence, years **^b^**			2.49 (3.35)
Family Present in Turkey			
Yes	53	34.6	
No	100	65.4	
Employment			
Employed	32	21.6	
Unemployed	116	78.4	
Income			
Stable	38	25.0	
Unstable	114	75.0	
Access to a Regular Source of Health Care			
Access	51	33.1	
No Access	103	66.9	
Short Form/SF-8			
Physical Health Status-Physical Component Summary/PCS **^c^**			43.38 (9.91)
Mental Component Score-Mental Component Summary/MCS **^d^**			38.87 (10.57)
Afghan Symptoms Checklist/ASCL **^e^**			59.81 (21.52)
Post-Migration Living Difficulties/PMLDs			
PMLD-1 (Conditions of Extreme Precarity) **^f^**			20.20 (10.17)
PMLD-2 (Asylum Difficulties) **^g^**			4.65 (4.78)
PMLD-3 (Employment-Related Problems) **^h^**			6.95 (4.09)
PMLD-4 (Access to Medical and Social Services) **^i^**			6.84 (5.53)
PMLD-5 (Marginalization and Family-Related Stressors) ^j^			8.44 (4.20)

Note. **^a^** Range: 18–57, **^b^** Range: <1–18 years, **^c^** Range: 15.58 (low physical health status)–63.53 (high physical health status), **^d^** Range: 13.55 (low mental health status)–61.54 (high physical health status), **^e^** Range: 23–115, **^f^** Range: 0 (not a problem at all)–36 (a very serious problem), **^g^** Range: 0 (not a problem at all)–16 (a very serious problem), **^h^** Range: 0 (not a problem at all)–12 (a very serious problem), **^i^** Range: 0 (not a problem at all)–16 (a very serious problem), **^j^** Range: 0 (not a problem at all)–12 (a very serious problem).

**Table 2:** Logistic regression analysis showing associations between selected determinants and utilization of general practitioner and outpatient specialist services.

Determinants	Primary Care Physician (Two Months)			Outpatient Specialist (Two Months)		
	Predisposing Factors	Predisposing and Enabling Factors	Predisposing, Enabling, and Need Factors	Predisposing Factors	Predisposing and Enabling Factors	Predisposing, Enabling, and Need Factors
	OR ^a^ CI (95%)	OR ^a^ CI (95%)	OR ^a^ CI (95%)	OR ^a^ CI (95%)	OR ^a^ CI (95%)	OR ^a^ CI (95%)
Predisposing Factors						
Gender **^b^**	3.088 * (1.1, 8.4)	3.984 * (1.2, 15.5)	7.777 ** (1.9, 32.5)	0.989 (0.30, 3.1)	0.693 (0.19, 2.6)	0.812 (0.18, 3.8)
Enabling Factors						
Family Presence in Turkey **^c^**		1.091 (0.39, 3.1)	1.253 (0.39, 4.0)		**2.685 ^†^ (0.99, 7.3)**	4.883 * (1.4, 16.6)
Income **^d^**		6.269 *** (2.3, 17.3)	4.182 * (1.2, 14.7)		3.790 * (1.4, 10.4)	1.703 (0.48, 6.0)
Access Regular Source **^e^**		1.818 (0.67, 5.0)	1.596 (0.55, 4.7)		**2.767 ^†^ (1.0, 7.5)**	2.264 (0.76, 6.8)
Need Factors						
Physical Health Status			0.941 (0.87, 1.0)			0.991 (0.92, 1.1)
Mental Health Status			1.025 (0.95, 1.1)			0.938 (0.87, 1.0)
Distress Symptoms			0.988 (0.95, 1.0)			0.947 * (0.90, 0.99)
PMLD-1 (Precarity)			**0.905 ^†^ (0.81, 1.0)**			0.926 (0.82, 1.0)
PMLD-2 (Asylum Difficulties)			1.202 * (1.0, 1.4)			1.359 ** (1.1, 1.7)
PMLD-3 (Employment-Related Problems)			1.048 (0.87, 1.3)			**0.834 ^†^ (0.68, 1.0)**
PMLD-5 (Marginalization and Family-Related Stressors)			0.945 (0.80, 1.1)			1.014 (0.85, 1.2)
Pseudo R^2^	0.048	0.266	0.380	0.000	0.253	0.429

Note. *n* = 130 due to listwise deletion; **^a^** OR: Odds ratios are adjusted for all other variables in each model, Reference categories: **^b^** males, **^c^** family not present, **^d^** unstable income, **^e^** No access; **^†^** *p* < 0.10, * *p* < 0.05, ** *p* < 0.01, *** *p* < 0.001.

**Table 3:** Logistic regression analysis showing association between selected determinants and utilization of prescription and nonprescription medications.

Determinants		Prescription Medications (14 Days)			Nonprescription Medications (14 Days)		
		Predisposing Factors	Predisposing and Enabling Factors	Predisposing, Enabling, and Need Factors	Predisposing Factors	Predisposing and Enabling Factors	Predisposing, Enabling, and Need Factors
		OR ^a^ CI (95%)	OR ^a^ CI (95%)	OR ^a^ CI (95%)	OR ^a^ CI (95%)	OR ^a^ CI (95%)	OR ^a^ CI (95%)
Predisposing Factors							
	Gender **^b^**	0.988 (0.33, 3.0)	0.600 (0.18, 2.1)	0.749 (0.20, 2.9)	0.881 (0.32, 2.4)	1.071 (0.37, 3.1)	1.110 (0.34, 3.6)
Enabling Factors							
	Family Presence in Turkey **^c^**		3.167 * (1.3, 8.0)	4.780 ** (1.7, 13.7)		0.641 (0.28, 1.5)	0.534 (0.21, 1.4)
	Income **^d^**		2.715 * (1.0, 7.1)	**3.148 ^†^ (0.92, 10.8)**		1.059 (0.44, 2.5)	2.562 (0.83, 7.9)
	Access Regular Source **^e^**		2.692 * (1.1, 6.7)	**2.045 ^†^ (0.77, 5.4)**		1.138 (0.50, 2.6)	1.333 (0.52, 3.4)
Need Factors							
	Physical Health Status			1.005 (0.95, 1.1)			0.953 (0.91, 1.0)
	Mental Health Status			0.956 (0.89, 1.0)			0.992 (0.94, 1.0)
	Distress Symptoms			**0.964 ^†^ (0.93, 1.0)**			**0.987 ^†^ (0.96, 1.0)**
	PMLD-1 (Precarity)			0.928 (0.84, 1.0)			1.066 (0.99, 1.2)
	PMLD-2 (Asylum Difficulties)			1.227 * (1.0, 1.4)			0.989 (0.90, 1.1)
	PMLD-3 (Employment-Related Problems)			1.054 (0.88, 1.3)			1.045 (0.90, 1.2)
	PMLD-5 (Marginalization and Family-Related Stressors)			1.102 (0.94, 1.3)			0.974 (0.85, 1.1)
	Pseudo R^2^	0.000	0.234	0.356	0.001	0.013	0.175

Note. *n* = 130 due to listwise deletion; **^a^** OR: Odds ratios are adjusted for all other variables in each model, Reference categories: **^b^** males, **^c^** family not present, **^d^** unstable income, **^e^** No access; **^†^** *p* < 0.10, * *p* < 0.05, ** *p* < 0.01.
